# Changes to Public Health Surveillance Methods Due to the COVID-19 Pandemic: Scoping Review

**DOI:** 10.2196/49185

**Published:** 2024-01-19

**Authors:** Emily C Clark, Sophie Neumann, Stephanie Hopkins, Alyssa Kostopoulos, Leah Hagerman, Maureen Dobbins

**Affiliations:** 1 National Collaborating Centre for Methods and Tools Hamilton, ON Canada; 2 School of Nursing McMaster University Hamilton, ON Canada

**Keywords:** public health, surveillance, digital surveillance, COVID-19, screening, infodemiology, big data, mobility tracking, wastewater, ethics, decision making, public health surveillance

## Abstract

**Background:**

Public health surveillance plays a vital role in informing public health decision-making. The onset of the COVID-19 pandemic in early 2020 caused a widespread shift in public health priorities. Global efforts focused on COVID-19 monitoring and contact tracing. Existing public health programs were interrupted due to physical distancing measures and reallocation of resources. The onset of the COVID-19 pandemic intersected with advancements in technologies that have the potential to support public health surveillance efforts.

**Objective:**

This scoping review aims to explore emergent public health surveillance methods during the early COVID-19 pandemic to characterize the impact of the pandemic on surveillance methods.

**Methods:**

A scoping search was conducted in multiple databases and by scanning key government and public health organization websites from March 2020 to January 2022. Published papers and gray literature that described the application of new or revised approaches to public health surveillance were included. Papers that discussed the implications of novel public health surveillance approaches from ethical, legal, security, and equity perspectives were also included. The surveillance subject, method, location, and setting were extracted from each paper to identify trends in surveillance practices. Two public health epidemiologists were invited to provide their perspectives as peer reviewers.

**Results:**

Of the 14,238 unique papers, a total of 241 papers describing novel surveillance methods and changes to surveillance methods are included. Eighty papers were review papers and 161 were single studies. Overall, the literature heavily featured papers detailing surveillance of COVID-19 transmission (n=187). Surveillance of other infectious diseases was also described, including other pathogens (n=12). Other public health topics included vaccines (n=9), mental health (n=11), substance use (n=4), healthy nutrition (n=1), maternal and child health (n=3), antimicrobial resistance (n=2), and misinformation (n=6). The literature was dominated by applications of digital surveillance, for example, by using big data through mobility tracking and infodemiology (n=163). Wastewater surveillance was also heavily represented (n=48). Other papers described adaptations to programs or methods that existed prior to the COVID-19 pandemic (n=9). The scoping search also found 109 papers that discuss the ethical, legal, security, and equity implications of emerging surveillance methods. The peer reviewer public health epidemiologists noted that additional changes likely exist, beyond what has been reported and available for evidence syntheses.

**Conclusions:**

The COVID-19 pandemic accelerated advancements in surveillance and the adoption of new technologies, especially for digital and wastewater surveillance methods. Given the investments in these systems, further applications for public health surveillance are likely. The literature for surveillance methods was dominated by surveillance of infectious diseases, particularly COVID-19. A substantial amount of literature on the ethical, legal, security, and equity implications of these emerging surveillance methods also points to a need for cautious consideration of potential harm.

## Introduction

Decision-making in public health draws on many sources of evidence. The best available research evidence, local health issues and context, current community and political climate, and evidence for existing public health resources all contribute to an evidence-informed decision-making approach [1].

Population-specific surveillance data provides critical evidence to inform decision makers on contextually relevant health issues. Public health surveillance data have been used to directly inform policies like animal importation laws; food recalls due to contaminated food; and communication to the public, such as promoting awareness of emerging environmental threats such as Lyme disease [2,3].

Public health surveillance plays a vital role in decision-making for maintaining and promoting population health. While surveillance programs are most well-known for tracking infectious disease outbreaks, programs also include screening for noncommunicable diseases. In Canada, public health surveillance occurs at national, provincial or territorial, regional, and municipal levels. At the national level alone, nearly 30 surveillance programs, networks, and systems operate to monitor disease and illness across Canada—such as the National Enteric Surveillance Program which monitors the incidence of foodborne illness, and FluWatch, which reports on the spread of influenza [4,5]. There are also regional and local surveillance processes that collect data, including the Ontario integrated Public Health Information System, which allows for reporting and surveillance of several diseases including enteric illness and influenza [6]. The surveillance data collected through these programs inform public health planning, programming, and response.

The COVID-19 pandemic and subsequent public health response led to a pivot in public health priorities. As the urgency to understand and respond to COVID-19 transmission grew, many public health teams prioritized pandemic response including the redeployment of many staff to contact tracing and eventually mass vaccination. For example, over 500 contact tracers were recruited in Ontario alone over a 2-month period in late 2020 [7], and, health care workers in British Columbia were hired or redistributed to form the COVID-19 Immunize BC Operations Centre to coordinate the province’s vaccine response [8]. Non–COVID-19 disease surveillance programs were impacted by emergency public health measures as well as reallocation of resources. Measures including physical distancing and service shutdowns impacted existing programs that were not considered essential, such as cancer screening programs that were paused or deferred during the height of the pandemic [9,10].

Access to “smart” or networked technology has become ubiquitous both in and outside of public health, with nearly 90% of Canadians owning a smartphone and 75% of Canadians using social media regularly [11-13]. The progression of public health surveillance methods has reflected a similar integration of technology, seeing many changes since the prominent SARS-CoV-1 epidemic in 2003. Newer surveillance tools include electronic health records, advancements in wastewater surveillance infrastructure and technology, and digital health surveillance [14]. Electronic health records are the technological response to navigate the complex Canadian health care system by digitally housing individuals’ full health histories [15]. Digitization of records also allows for centrally tracking infectious diseases that may enter the country or region [14]. Prior to the COVID-19 pandemic, wastewater surveillance had gained popularity for tracking infectious disease dynamics and tracing the origins of foodborne illness outbreaks [16,17]. As it is difficult to estimate the true burden of disease of foodborne illnesses, wastewater surveillance has proven to be an accurate and relatively inexpensive way of collecting health data [17,18]. Similarly, digital surveillance can use internet search data to track where and when disease outbreaks occur [19]. More individually, connection to WiFi and GPS tracking can provide access to millions of smartphone users’ locations, enabling accurate and specific tracking of outbreaks and other public health issues [20,21].

Despite advances that occurred prior to the COVID-19 pandemic, the need for improvement of public health surveillance capacity and methods has persisted in Canada and internationally [22-26]. Existing barriers in public health include low-quality data and obstacles to data sharing, as well as political and professional barriers such as incompatible technical lexicons and territorial borders [26]. This adds to the pertinence in investigating how the COVID-19 pandemic impacted public health surveillance and how it may continue to do so [27]. For example, the singular worldwide focus on COVID-19 allowed for an unprecedented dedication of resources (time, money, and intellect) to leverage pre-existing surveillance processes, creating the opportunity to apply these learnings to non–COVID-19 disease surveillance contexts [28]. The severity of the COVID-19 pandemic necessitated a further push in the development of innovative surveillance methods to track and manage SARS-CoV-2 transmission [29,30]. As a result, there have been marked improvements in the ability to predict, track, and respond to COVID-19 outbreaks in Canada and around the world [31-34].

However, there is a need for ethical consideration to be at the forefront of these novel surveillance applications [18,35]. Although the ubiquity of personal technology like smartphones presents convenient and cost-effective disease surveillance opportunities, concerns around privacy and stigma through potential applications have emerged [36-39]. Digital surveillance tools that capture data through personal devices such as smartphones have the ability to capture personally identifiable information including race-based and health history data [35]. As digital surveillance itself is fairly novel there are few regulatory and legal frameworks in place to protect the misuse of this data [35]. Transparency, in terms of what information is being collected and how it is being used, is an additional ethical consideration that has yet to be fully addressed [38].

The National Collaborating Centre for Methods and Tools’ Rapid Evidence Service [40] was established at the onset of the COVID-19 pandemic to conduct rapid reviews on priority public health topics. This review was requested of the rapid evidence service by the Public Health Agency of Canada to understand how public health surveillance was changed by the COVID-19 pandemic, resulting in a knowledge synthesis to inform future decision-making. This rapid scoping review investigates the question: What is known about changes to public health methods at the population level for governments globally due to the COVID-19 pandemic?

## Methods

### Study Design

A scoping review capturing literature on changes to public health surveillance in response to the COVID-19 pandemic was conducted and is reported according to the PRISMA-ScR (Preferred Reporting Items for Systematic Reviews and Meta-Snalyses extension for Scoping Reviews) [41]. Review methods were based on previously described rapid review methods [40], with adaptations to a scoping review framework [42]. Inclusion and exclusion criteria were defined based on population, concept, and context criteria rather than population, intervention or exposure, comparison, and outcome criteria. Findings from included studies were not extracted and studies were not assessed for study quality, since the review question focused on identifying a breadth of evidence, rather than individual study findings. Data were analyzed for overall trends and presented visually.

### Information Sources and Search Strategy

The search was conducted on January 25, 2022. The following databases were searched: Medline, Embase, Emcare, Global Health Database, PsycInfo, MedRxiv, COVID-19 living overview of evidence, Web of Science, and the World Health Organization’s global literature on coronavirus disease. Each data were searched using combinations of the terms “monitor,” “surveillance,” and “coronavirus.” Additionally, international government websites were hand-searched for relevant gray literature. The full search strategy is included in Multimedia Appendix 1 [14,28-30,32-34,36,43-274].

### Eligibility Criteria

Literature on population-level surveillance was included if surveillance was established in response to the COVID-19 pandemic, or directly impacted by the COVID-19 pandemic, through reprioritization of resources or public health restrictions. Studies and reports published between March 1, 2020 and January 25, 2022, were included. While the first cases of COVID-19 were identified in December 2019, the search was timeframe limited to March 2020 or after to allow for literature to capture changes in surveillance and reduce the overall results set for this rapid scoping review. English- or French-language, peer-reviewed sources, and sources published ahead of print before peer review were included. Gray literature was also included. Detailed criteria for inclusion and rationale are outlined in Textbox 1.

**Inclusion and exclusion criteria for rapid scoping review of changes to public health surveillance methods during the COVID-19 pandemic March 2020-January 2022.**

**Population:**

**Inclusion criteria**
  National, provincial, territorial, state, and regional population-level surveillance.
**Exclusion criteria**
  Surveillance of an individual.
**Rationale**
  To inform the planning of population-level surveillance programs and systems.

**Concept:**

**Inclusion criteria**
  Surveillance programs and systems that have been directly affected by the COVID-19 pandemic.
**Exclusion criteria**
  Surveillance programs and systems not directly impacted by the COVID-19 pandemic.
**Rationale**
  To explore shifts in surveillance approaches in response to the COVID-19 pandemic.

**Context:**

**Inclusion criteria**
  COVID-19 pandemic: March 1, 2020, to January 25, 2022.
**Rationale**
  To capture changes in surveillance methods due to the COVID-19 pandemic and reduce overall scope.

Studies were screened using DistillerSR software (DistillerSR). Titles and abstracts of retrieved studies were screened by a single reviewer (SN). Full texts of included studies were screened by a second reviewer (ECC) and reviewed by a third (MD). The screening was not completed in duplicate to maintain a rapid timeline.

### Data Extraction

Data extraction was completed by a single reviewer (ECC or SH). Details on the surveillance method (eg, wastewater sampling and digital data collection), as well as surveillance subjects (eg, COVID-19 and population mental health) for each included source were extracted. The type of evidence source was also extracted (eg, syntheses, single study, and opinion).

Studies, critical reviews, editorials, and newspaper articles commenting on the ethics of surveillance methods that were captured by the search were briefly summarized. Details of the method of surveillance and the focus of the ethical implications (ie, ethics, security, legality, and equity) were extracted. Consistent with a scoping review approach, quality assessments of included studies were not completed.

### Data Analysis

Sources were grouped by surveillance method and surveillance subject to identify overall trends in the literature. Papers presenting ethical implications of novel approaches to surveillance were grouped by the paper’s focus.

Findings are presented as overall trends in the literature [42]. Results are discussed narratively and summarized in tables. To visually depict overall findings, a Sankey diagram was generated pairing surveillance methods and surveillance subjects, using the software SankeyMATIC (SankeyMATIC). Sankey diagrams can represent the flow of material or ideas through a system. The scale of each section of a Sankey diagram is proportional to the amount of material flowing between stages [43,275-277].

### Peer Reviewers

Involving patient and public partners in rapid reviews has emerged as a valuable addition to review methodology. Gaining perspectives from patients and public partners can help ensure that rapid reviews meet knowledge users’ needs and are relevant for decision-making [278,279]. The Cochrane collaboration, a world leader in systematic review methodology, has identified consumer involvement throughout the process of evidence production and dissemination as a vital component [280,281]. Given the more technical focus of this review, rather than seeking partners with lived experience from the general public, epidemiologists who worked closely with surveillance efforts during the COVID-19 pandemic were invited as peer reviewers through organizational connections. Partners were invited to provide feedback on review findings based on lived experiences working in the field. Specifically, they were asked to review the Executive Summary and reflect on their own experiences with shifts in public health surveillance during the COVID-19 pandemic, and to highlight gaps in the literature. Their perspectives are not presented as a source of objective data alongside review findings, but rather as contextualizing perspectives of the objective data found by the review. Both partners provided written feedback that informed the review’s conclusions.

## Results

### Overview

**Figure 1:**
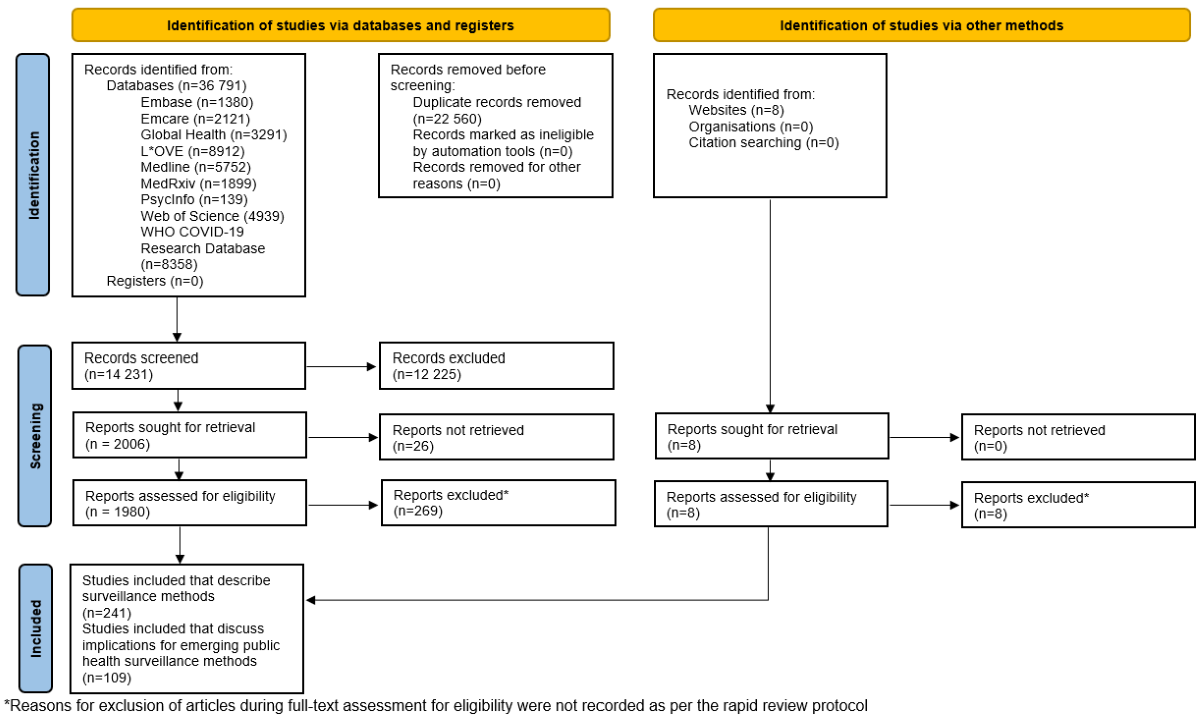
PRISMA-ScR (Preferred Reporting Items for Systematic Reviews and Meta-Analyses extension for Scoping Reviews) flow diagram of studies included in this scoping review of changes to public health surveillance during the COVID-19 pandemic from March 2020 through January 2022.

Database searching retrieved 36,791 records and scanning of key government websites retrieved 8 records. After removing duplicates, 14,238 records were screened by title and abstract, resulting in 1980 reports for full-text review. Of those 1980 records, 241 papers that described surveillance methods and 108 papers that discussed the implications of changes to public health surveillance programs from ethical, security, legal, and equity perspectives were included in the scoping review and underwent data extraction. See Figure 1 for a PRISMA-ScR flowchart illustrating the paper search and selection process.

### Study Characteristics

Of the 241 papers that described public health surveillance methods, 80 were review papers or evidence syntheses and 161 were single studies. Literature was dominated by reports of COVID-19 surveillance (n=187), but other infectious diseases were also represented, including other respiratory and viral pathogens (n=3), and gastrointestinal (n=3), sexually transmitted (n=2) and zoonotic infections (n=4). There were several papers reporting on surveillance related to immunization, including vaccine efficacy (n=1), vaccine safety monitoring (n=4), and vaccine hesitancy (n=4). Some papers reported on cancer screening (n=5) during the COVID-19 pandemic. Surveillance of mental health (n=11) and substance use (n=4) were also described. One paper described surveillance of healthy nutrition (n=1) and other papers described surveillance of maternal and child health (n=3). Papers described monitoring of antimicrobial resistance (n=2). Several papers described monitoring misinformation on the web (n=6). Table 1 summarizes the number of syntheses and single studies by public health surveillance subject, organized by public health topic area. A detailed list of included studies is available in Table S1 in Multimedia Appendix 1 [14,28-30,32-34,36,43-274].

**Table 1:** Numbers of syntheses and single studies of public health surveillance method changes among public health surveillance subjects from March 2020 through January 2022.

Public health topic area and surveillance subject		Syntheses, n	Single studies, n
**Antimicrobial stewardship**			
	Antimicrobial resistance	1	1
**Chronic diseases and conditions**			
	Cancer screening	1	4
**Health promotion**			
	Nutrition	1	0
	Maternal and child health	0	3
**Immunization**			
	Vaccine efficacy	0	1
	Vaccine safety	1	3
	Vaccine hesitancy	0	4
**Infectious diseases**			
	COVID-19	67	120
	Other respiratory pathogens (general)	1	0
	Other viral pathogens (general)	1	1
	Vaccine-preventable diseases	0	1
	Gastrointestinal infections	1	2
	Sexually transmitted infections	1	1
	Zoonoses	1	3
**Mental health and substance use**			
	Mental health	2	9
	Substance use	1	3
**Health communication**			
	Misinformation	1	5

Singles studies were conducted in the United States (n=51), Europe (n=27), East Asia (n=17), United Kingdom (n=9), Canada (n=5), South America (n=4), Africa (n=3), Australia (n=3), South Asia (n=2), and Russia (n=1). A total of 12 studies were conducted across several countries, and 27 studies were conducted globally. Global studies were mostly digital surveillance studies using global data sets.

Of the 80 included evidence syntheses, the majority were nonsystematic literature reviews (n=61). Syntheses also included systematic reviews (n=10), scoping reviews (n=7), 1 rapid review, and 1 guideline.

### Surveillance Methods

**Figure 2:**
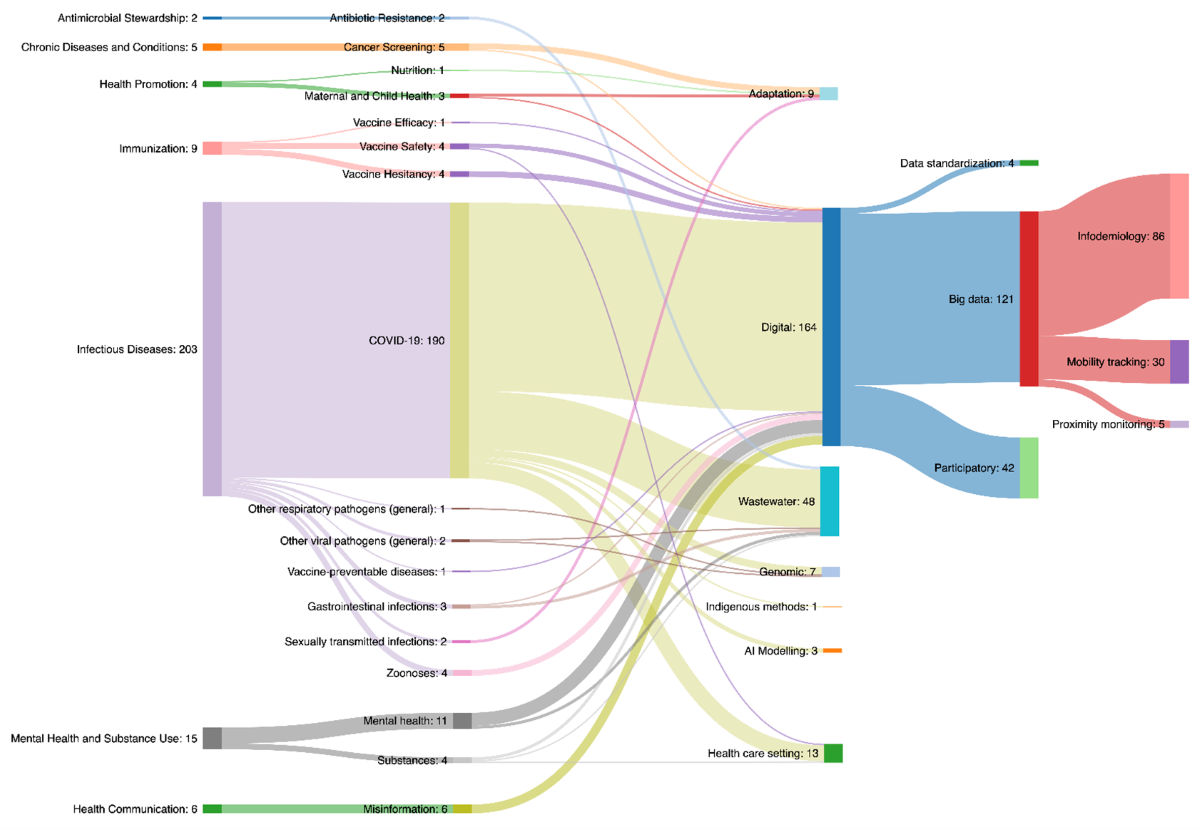
Sankey diagram representing the number of included papers for various surveillance methods used for the target surveillance subjects, listed by public health topic area, for the time period from March 2020 to January 2022. Please note that the numbers for each surveillance method may not reflect the total number of resources in this report, as some syntheses discuss more than 1 surveillance method.

The Sankey diagram in Figure 2 illustrates the number of included papers that report on the various surveillance methods used for the target surveillance subjects, listed by public health topic area.

Most of the papers on surveillance of COVID-19 transmission described digital surveillance (eg, 130 papers on the use of big data through mobility tracking and infodemiology and 40 papers on wastewater epidemiology). Other methods for monitoring COVID-19 transmission were designed to supplement limited COVID-19 testing capacity, including analysis of data from various health care settings, for example, hospitals and blood banks (n=11). Strategies to track COVID-19 transmission and detect new SARS-CoV-2 variants through genome sequencing were also described (n=5).

Literature for select public health surveillance functions that operated prior to the COVID-19 pandemic, such as cancer screening, child health assessments, and sexually transmitted infection screening, indicated that these functions adapted to using technology for remote screening in response to mobility restrictions and physical distancing. For example, 2 studies described videoconferencing for remote autism screening in young children, and another study described a pilot program for remote sexually transmitted infection self-testing under the supervision of public health staff through videoconference.

Other public health surveillance programs took approaches similar to COVID-19 surveillance. For example, digital surveillance technologies were applied to monitor population mental health (n=8), misinformation (n=5), and zoonoses (n=2). In addition to COVID-19, wastewater surveillance was used to monitor antibiotic resistance (n=2), mental health (n=2), substance use (n=1), as well as other infectious diseases.

Artificial intelligence (AI) was used to augment digital surveillance of COVID-19 transmission (n=29), immunization (n=2), substance use (n=2), and misinformation (n=3; Table 2). Most of these used AI to comb through population-level cell phone mobility data, social media content, or search engine queries for indications of COVID-19 transmission. AI was also used to analyze social media content and search engine queries for indications of vaccine hesitancy, substance use, and misinformation. One paper described using AI to analyze global wastewater data for COVID-19 transmission pathways.

**Table 2:** Summary of the number of papers for each new method of public health surveillance from March 2020 through January 2022.

Method				Number of included papers		Number using artificial intelligence
Adaptation to existing practice				9		0
AI-driven modeling				3		3
**Digital surveillance**				163		36
	Data standardization			4		0
	Participatory			39		4
	**Big data**			131		32
		Mobility tracking	36		12	
		Proximity monitoring	5		0	
		Infodemiology	86		22	
Health care setting				11		0
Indigenous methods				1		0
Wastewater				48		1

Several studies (n=4) described the development of standardized, digitized data repositories to enable interjurisdictional surveillance [44-47]. One of these described standardized electronic reporting for cancer screening to support central cancer registries [44]. A literature review described distributed data networks to monitor COVID-19 vaccine safety issues [45]. Two papers described data federation technologies for infectious diseases other than COVID-19. A literature review by Baker et al [46] described the standardization of data for malaria, and a pilot study by Colella et al [47] described the development of a biorepository platform to monitor animal-to-human disease transmission. These papers emphasized the interoperability of data systems to facilitate large-scale surveillance of public health issues.

One study used Indigenous methods to monitor COVID-19 through a community-focused COVID-19 surveillance program [48]. In this study, the Hopi Tribe, a sovereign nation in the United States, collaborated with the US Centers for Disease Control and Prevention to deploy community health representatives to every household in 2 Hopi villages [48]. Community health representatives screened household members for COVID-19 symptoms, exposures, and provided education, all in a culturally safe manner [48]. No other studies using Indigenous methods were found.

### Ethical Implications

In addition to papers describing public health surveillance methods, 108 papers exploring the implications of changes to public health surveillance programs during the COVID-19 pandemic, from ethical, legal, data security, and equity perspectives were retrieved. These papers focused on digital surveillance (n=104) or wastewater surveillance (n=4) and discussed potential consequences of surveillance in terms of ethics (n=80), data security (n=28), legality (n=28), and equity (n=21), noting that some papers covered more than 1 topic area. A detailed list of included studies is available in Table S2 in Multimedia Appendix 2 [37-39,282-386].

Aside from 2 systematic reviews, most of the papers discussing the implications of surveillance were critical literature reviews, conducted nonsystematically and written to express a clear opinion or bias (n=71). There were 36 editorials that did not cite or infrequently cited other literature and were published in academic journals (n=29) or newspapers (n=7).

### Perspectives From Public Health Epidemiologists

Two public health epidemiologists who worked in Ontario during the COVID-19 pandemic provided feedback on the findings of this review. They suggested that nearly every surveillance system or program was impacted by the COVID-19 pandemic; some paused due to resource reallocation. Several key metrics that were previously reliable indicators became difficult or not possible to interpret due to the impact of COVID-19. These changes may not be captured in academic literature featured in this review, as many public health professionals working during the pandemic simply did not have the time, energy, or academic support to publish their work.

Epidemiologists shared that the overall approach to surveillance of COVID-19 also changed over the course of the pandemic. While case counts were an early key indicator, there has been a general shift to measures such as hospital and intensive care unit occupancy, reflecting the change in priority from eliminating COVID-19 altogether to reducing and managing the health care system burden.

It was also suggested by epidemiologists that while data for equity has been limited, some governments have made efforts to improve measures of equity. For example, in 2020 the Ontario government amended the Health Protection and Promotion Act and augmented the provincial case and contact management tool to allow for the collection of social determinants (eg, race and income) for COVID-19 cases. Similar changes were made for COVID-19 vaccine surveillance data.

It was suggested that substantial resources are required to restore public health surveillance systems during pandemic recovery and improvements can be made by leveraging or investing in better data.

## Discussion

### Principal Findings

This scoping review provides a comprehensive overview of changes to public health surveillance methods for programs, systems, and strategies at the population level implemented since early 2020. The review includes 241 papers, including 80 evidence syntheses and 161 single studies describing surveillance methods, as well as 108 papers discussing the implications of changes to public health surveillance programs from ethical, security, legal, and equity perspectives. A Sankey diagram (Figure 2) illustrates the dominance of COVID-19 surveillance in this literature, particularly through digital surveillance strategies.

The heavy focus on COVID-19 in the surveillance literature reflects public health’s drastic reallocation of resources to the pandemic response [387,388]. As nearly all countries of the world entered some form of public health lockdown, unique research opportunities presented themselves [389,390]. Health researchers redirected efforts to contribute to global focus to understand and mitigate COVID-19 transmission and harm [391]. Additionally, academic journals expedited the publication process for papers on COVID-19 [392,393]. While other public health subjects, such as maternal and child health, chronic diseases, and infectious diseases other than COVID-19 were also represented in the surveillance literature, these subjects were largely overshadowed by COVID-19. As suggested by the public health epidemiologists included in this review, those working in surveillance for other public health topics likely did not have time or resources to publish during the pandemic.

Within the COVID-19 surveillance literature, a substantial proportion of papers discussed digital forms of surveillance. This was largely using sources of big data, such as social media posts, search engine queries, and mobility tracking. Prior to the COVID-19 pandemic, there had been a steady rise in publications on big data, as such the field was sufficiently established to contribute to COVID-19 tracking and analysis at the onset of the pandemic [394,395]. Publications were largely proof-of-concept, demonstrating if and how big data analysis correlated with levels of COVID-19 infections seen in communities. Following the interest and advancements in the analysis of big data, these methods are likely to continue to see novel applications in public health.

Wastewater surveillance emerged as a dominant method in the COVID-19 surveillance literature. Wastewater testing is an attractive option for disease surveillance as it is a cost-effective method to monitor populations without requiring active participation or invasive testing procedures [396-398]. Similar to the literature for big data analysis, much of the literature on wastewater provided proof of concept by correlating wastewater detection of the SARS-CoV-2 virus with reported levels of COVID-19 in the community. The impact of wastewater surveillance on public health decision-making is likely to grow [399]. Recently, the detection of vaccine-derived poliovirus in wastewater in New York City led to the addition of the United States to the World Health Organization’s list of countries with polio transmission [400]. To expand coverage across Canada’s population, the Public Health Agency of Canada invested in a pan-Canadian wastewater surveillance network for COVID-19, in collaboration with other federal departments; provincial, territorial, and municipal governments; and academic institutions [401]. While the focus for wastewater surveillance was largely on COVID-19, advances in wastewater monitoring capacity allowed for progress in wastewater monitoring of other conditions and issues, such as mental health and substance use. Given the investment in wastewater epidemiology and advancements in its applications, it is likely to emerge as a key indicator for public health post the pandemic.

In the context of COVID-19 pandemic lockdowns and physical distancing, it is not surprising that some public health services, such as childhood nutrition, cancer screening, and sexual health, were adapted to remote provision of services. While several papers describing the implementation of remote services were included in this review, it is likely that these papers represent only a small sample of public health efforts to continue service provision during the pandemic. As indicated by the public health epidemiologists consulted for this review, many changes to existing public health surveillance programs are likely not published in the academic literature, due to the demands on public health professionals’ time.

The COVID-19 pandemic also highlighted the importance of interjurisdictional data systems which can enable timely access to data across jurisdictional boundaries to inform public health functions. Several papers included in this review explored standardization of data to enhance the public health response, for monitoring zoonoses [46,47] or rare vaccine-related harms [45] at a population level. Solutions to the current limitations of data interoperability must be implemented in future public health surveillance efforts to improve access to essential data in real-time that can inform decision-making within critical health organizations and governing bodies.

While technology has enabled many advancements in public health surveillance, the findings of this scoping review also demonstrate the alarm caused by these changes. The ethical implications of digital surveillance, in particular, were discussed in 80 included papers. Since smartphones and the internet of things (a system of interrelated computing devices to connect people or things identified by unique identifiers), have only become widely used relatively recently, with the potential consequences of their use in public health surveillance not being fully realized yet. The COVID-19 pandemic accelerated the development and testing of digital surveillance, and the literature reflects that experts in these fields have cause for concern. The relative lack of literature discussing the impact of changes in public health surveillance on equity is also concerning. The pandemic exacerbated existing inequities, and care must be taken so that these emerging methods do not further widen equity gaps [402-406]. Data collection and analysis for equity-deserving populations must be a priority as the identification of inequities allows for mitigation. However, these data must be collected and handled with care and respect to avoid potential harm and stigmatization [407,408].

### Limitations

Two public health epidemiologists who contributed their perspectives to this review raised that many changes to surveillance are not captured in the literature, primarily due to the lack of time and academic support for public health in publishing. This is an important limitation of this review, as the full scope of changes to public health surveillance is likely not represented. Academic partnerships and support for public health can help share innovations [409].

The peer perspectives shared in this review are also limited to 2 epidemiologists based in Canada, which may not fully represent the perspectives of those involved in public health surveillance worldwide. However, the epidemiologists involved provided contextual insights to the findings of this review.

This review is also limited to papers from March 2020 to January 2022. Studies published after January 2022 may be more likely to capture changes to public health surveillance that are not directly related to COVID-19. For example, examples of wastewater surveillance of pathogens other than SARS-CoV-2 may not have been captured by this search.

As a scoping review of the literature for changes to public health surveillance methods during the COVID-19 pandemic, there are limitations to the overall conclusions that can be supported by this review’s findings. Given the scoping review approach, findings for the effectiveness of surveillance methods and the quality of included papers were not evaluated. A systematic analysis of included papers, including the extraction of outcome data, rigorous quality assessment of included papers, and judgments of the certainty of evidence, is strongly recommended when considering the potential future implementation of any of the surveillance methods discussed in this report.

### Conclusions

A considerable body of literature emerged related to advances in surveillance during the COVID-19 pandemic. Some areas have seen much more literature, such as wastewater epidemiology, while others remain sparse, such as health equity for racialized populations. Possible next steps include conducting systematic reviews in areas for which multiple studies have been identified. In other areas with sparse literature, more research is needed to advance surveillance in the postpandemic era. A large volume of papers expressing concerns about the potential ethical, legal, security, and equity implications of emerging surveillance methods indicates a need for a thorough assessment of the potential effects of large-scale implementation of these surveillance methods.

## Appendix

Multimedia Appendix 1 Included articles that describe public health surveillance methods.

Multimedia Appendix 2 Included articles that describe implications of emerging public health surveillance methods.

Multimedia Appendix 3 PRISMA-ScR Checklist.

## Abbreviations

AI: artificial intelligence
PRISMA-ScR: Preferred Reporting Items for Systematic Reviews and Meta-Analyses extension for Scoping Reviews

## Data Availability

The data sets used and analyzed in the conduct of this review are available from the corresponding authors on reasonable request.
