# Supplementary material for: Changes to Public Health Surveillance Methods Due to the COVID-19 Pandemic: Scoping Review
Source: JMIR Public Health Surveill. 2024 Jan 19;10:e49185. doi: 10.2196/49185 (PMC10837764; doi:10.2196/49185)
Supplement: Multimedia Appendix 1 [file publichealth_v10i1e49185_app1.docx]

**Table A1: Included articles that describe public health surveillance methods**

|  | **Study** | **Public Health subject** | **Location** | **Surveillance method** | **Level of evidence** | **Study design** |
| --- | --- | --- | --- | --- | --- | --- |
|  |  |  |  |  |  |  |
| **Antimicrobial stewardship** |  |  |  |  |  |  |
|  | Pruden, 2021 (76) | Antimicrobial resistance | Global | Wastewater | Synthesis | Non-systematic literature review |
|  | Riquelme, 2021 (77) | Antimicrobial resistance | International: India, Hong Kong, Philippines, Sweden, Switzerland, USA | Wastewater | Single study | Cross-sectional |
| **Chronic diseases and conditions** |  |  |  |  |  |  |
|  | Mehta, 2021 (78) | Cancer screening | Global | Adaptation | Synthesis | Non-systematic literature review |
|  | Maresca, 2021 (79) | Cancer screening | Grampian region, Scotland | Adaptation | Single study | Cohort |
|  | Jones, 2021 (47) | Cancer screening | USA | Digital: data standardization | Single study | Descriptive |
|  | Sinharay, 2020 (80) | Cancer screening | Cambridge, UK | Adaptation | Single study | Cross-sectional |
|  | Xiao, 2021 (81) | Cancer screening | Chicago, Illinois, USA | Adaptation | Single study | Cohort |
| **Health promotion** |  |  |  |  |  |  |
|  | UNICEF, 2020 (82) | Nutrition | Global | Adaptation | Synthesis | Guideline |
|  | Eapen, 2021 (83) | Maternal and child health | New South Wales, Australia | Digital: user input app | Single study | Randomized controlled trial |
|  | Maitre, 2021 (84) | Maternal and child health | Columbus, Ohio, USA | Adaptation | Single study | Time series |
|  | Conti, 2020 (85) | Maternal and child health | Tuscany, Italy | Adaptation | Single study | Descriptive |
|  | Varrelman, 2021 (86) | Vaccine efficacy | Gauteng, South Africa | Digital: user input app | Single study | Cohort |
|  | Dodd, 2021 (48) | Vaccine safety | Global | Digital: data standardization | Synthesis | Non-systematic literature review |
|  | Guedel, 2021 (87) | Vaccine safety | Basel, Switzerland | Digital: user input app | Single study | Cohort |
|  | Salter, 2020 (88) | Vaccine safety | Australia | Digital: user input app | Single study | Cohort |
|  | Schuemie, 2021 (89) | Vaccine safety | USA | Health care setting | Single study | Cohort |
|  | Beeferman, 2021 (90) | Vaccine hesitance | USA | Digital: infodemiology | Single study | Cross-sectional |
|  | Liew, 2021 (91) | Vaccine hesitance | Global | Digital: infodemiology using AI | Single study | Cross-sectional |
|  | Zhou, 2021 (92) | Vaccine hesitance | Global | Digital: infodemiology using AI | Single study | Time series |
|  | Rovetta, 2021 (93) | Vaccine hesitance | Italy | Digital: infodemiology | Single study | Cross-sectional |
| **Infectious diseases (non-COVID-19)** |  |  |  |  |  |  |
|  | Carr, 2021 (94) | Respiratory pathogens | Global | Genomic | Synthesis | Non-systematic literature review |
|  | Mohsin, 2021 (95) | Viral infections | Global | Genomic | Synthesis | Non-systematic literature review |
|  | Nieuwenhuijse, 2020 (96) | Viral infections | Global | Wastewater | Single study | Cross-sectional |
|  | Obeidat, 2020 (97) | Vaccine-preventable diseases | USA | Digital: infodemiology | Single study | Time series |
|  | Yuan, 2021 (98) | Gastrointestinal infections | New York and California, USA | Digital: infodemiology | Single study | Time series |
|  | Zahedi, 2021 (99) | Gastrointestinal infections | Global | Wastewater | Synthesis | Non-systematic literature review |
|  | Tedcastle, 2022 (100) | Gastrointestinal infections | UK | Wastewater | Single study | Time series |
|  | Rivera, 2021 (101) | STIs (HIV) | New York City, New York, USA | Telehealth | Single study | Descriptive |
|  | Kersh, 2021 (102) | STIs | USA | Adaptation | Synthesis | Non-systematic literature review |
|  | Baker, 2020 (49) | Zoonoses (Malaria) | Global | Digital: data standardization | Synthesis | Non-systematic literature review |
|  | Tizzani, 2021 (103) | Zoonoses (African swine fever) | Europe, Asia | Digital: infodemiology | Single study | Time series |
|  | Peterson, 2021 (14) | Zoonoses (Arboviral disease) | USA | Digital: infodemiology | Single study | Cross-sectional |
|  | Colella, 2021 (50) | Zoonoses | North and South America | Digital: data standardization | Single study | Descriptive |
| **Mental health and substance use** |  |  |  |  |  |  |
|  | Brogly, 2021 (104) | Mental health | London, Ontario, Canada | Digital: user input app | Single study | Cohort |
|  | Cohrdes, 2021 (105) | Mental health | Germany | Digital: infodemiology | Single study | Cross-sectional |
|  | Gimbrone, 2021 (106) | Mental health | New York City, New York, USA | Digital: infodemiology | Single study | Time series |
|  | Guntuku, 2020 (107) | Mental health | USA | Digital: infodemiology | Single study | Cohort |
|  | Keyes, 2021 (108) | Mental health | USA | Digital: infodemiology | Synthesis | Non-systematic literature review |
|  | Knipe, 2021 (109) | Mental health | UK | Digital: infodemiology | Single study | Time series |
|  | Lin, 2020 (110) | Mental health | International | Digital: infodemiology | Single study | Time series |
|  | Nason, 2021 (111) | Mental health | New Haven, Connecticut, USA | Wastewater | Single study | Time series |
|  | Sycinska-Dziarnowska, 2021 (112) | Mental health | Global | Digital: infodemiology | Single study | Time series |
|  | Tsao, 2021 (113) | Mental health | Global | Digital: infodemiology | Synthesis | Scoping review |
|  | Melchor-Martinez, 2021 (114) | Mental health | UK | Wastewater | Single study | Cross-sectional |
|  | Erickson, 2021 (115) | Substances | Global | Wastewater | Synthesis | Non-systematic literature review |
|  | Kilby, 2021 (116) | Substances | USA | Digital: infodemiology using AI | Single study | Time series |
|  | Lavertu, 2021 (117) | Substances | USA | Digital: infodemiology using AI | Single study | Time series |
|  | Slavova, 2020 (118) | Substances | Kentucky, USA | Health care setting | Single study | Time series |
| **Health Communication** |  |  |  |  |  |  |
|  | Hou, 2020 (119) | Misinformation | China | Digital: infodemiology | Single study | Time series |
|  | Mackey, 2020 (120) | Misinformation | Global | Digital: infodemiology using AI | Single study | Time series |
|  | Shahsavari, 2020 (121) | Misinformation | Global | Digital: infodemiology using AI | Single study | Cross-sectional |
|  | Shams, 2021 (122) | Misinformation | Global | Digital: infodemiology using AI | Single study | Descriptive |
|  | Storeng, 2021 (123) | Misinformation | Europe | Digital: infodemiology | Synthesis | Non-systematic literature review |
|  | Tan, 2021 (124) | Misinformation | Singapore | Digital: user input app | Single study | Cohort |
| **COVID-19** |  |  |  |  |  |  |
|  | Syrowatka, 2021 (125) | COVID-19 | Global | Modelling using AI | Single study | Modelling study |
|  | Kurita, 2020 (126) | COVID-19 | Japan | Modelling using AI | Single study | Modelling study |
|  | Ramchandani, 2020 (127) | COVID-19 | USA | Modelling using AI | Single study | Modelling study |
|  | Hansen, 2021 (128) | COVID-19 | Denmark | Digital: Big Data | Single study | Time series |
|  | Conrad, 2021 (129) | COVID-19 | Los Angeles, California, USA | Digital: Big Data | Single study | Modelling study |
|  | Zeng, 2021 (130) | COVID-19 | Hungary | Digital: Big Data | Single study | Time series |
|  | Szocska, 2021 (131) | COVID-19 | Memphis, Tennessee, USA | Digital: Big Data | Single study | Time series |
|  | Brakefield, 2020 (32) | COVID-19 | Global | Digital: Big Data | Single study | Modelling study |
|  | Sulyok, 2020 (132) | COVID-19 | Brazil | Digital: Big Data | Single study | Modelling study |
|  | De Oliveira, 2020 (133) | COVID-19 | Brazil | Digital: Big Data | Single study | Time series |
|  | Verhulst, 2021 (134) | COVID-19 | Global | Digital: Big Data | Synthesis | Non-systematic literature review |
|  | Alamo, 2021 (135) | COVID-19 | Global | Digital: Big Data | Synthesis | Non-systematic literature review |
|  | Nageshwaran, 2021 (136) | COVID-19 | International (Taiwan, South Korea, Hong Kong, Singapore) | Digital: Big Data | Synthesis | Non-systematic literature review |
|  | Ribeiro-Navarrete, 2021 (137) | COVID-19 | Global | Digital: Big Data | Synthesis | Systematic review |
|  | Kurian, 2020 (138) | COVID-19 | Global | Digital: Big Data | Synthesis | Non-systematic literature review |
|  | Saran, 2020 (139) | COVID-19 | Global | Digital: Big Data | Synthesis | Non-systematic literature review |
|  | Jalabneh, 2020 (36) | COVID-19 | Global | Digital: Big Data | Synthesis | Non-systematic literature review |
|  | Najah, 2020 (140) | COVID-19 | Global | Digital: Big Data | Synthesis | Non-systematic literature review |
|  | Sonn, 2020 (141) | COVID-19 | South Korea | Digital: Big Data | Synthesis | Non-systematic literature review |
|  | Abidemi Adeniyi, 2020 (142) | COVID-19 | Global | Digital: Big Data | Synthesis | Non-systematic literature review |
|  | Kishore, 2021 (143) | COVID-19 | India | Digital: Big Data | Single study | Time series |
|  | Zheng, 2021 (144) | COVID-19 | USA | Digital: Big Data | Single study | Modelling study |
|  | Aljumah, 2021 (145) | COVID-19 | Global | Digital: Big Data using AI | Single study | Modelling study |
|  | Ouyang, 2021 (34) | COVID-19 | Global | Digital: Big Data using AI | Single study | Cross-sectional |
|  | Ahmed, 2021 (146) | COVID-19 | Global | Digital: Big Data using AI | Single study | Cross-sectional |
|  | Wang, 2020a (147) | COVID-19 | China | Digital: Big Data using AI | Single study | Modelling |
|  | Budd, 2020 (148) | COVID-19 | Global | Digital: Big Data using AI | Synthesis | Non-systematic literature review |
|  | Abd-Alrazaq, 2021 (149) | COVID-19 | Global | Digital: Big Data using AI | Synthesis | Scoping review |
|  | Rana, 2020 (150) | COVID-19 | Global | Digital: Big Data using AI | Synthesis | Non-systematic literature review |
|  | Wang, 2020b (151) | COVID-19 | USA | Digital: Big Data using AI | Single study | Modelling |
|  | Kostkova, 2021 (152) | COVID-19 | Global | Digital: Big Data, infodemiology | Synthesis | Systematic review |
|  | Golinelli, 2020 (153) | COVID-19 | Global | Digital: Big Data, infodemiology and AI | Synthesis | Systematic review |
|  | Ahmed, 2022 (154) | COVID-19 | Global | Digital: Big Data, infodemiology with AI | Synthesis | Non-systematic literature review |
|  | Radanliev, 2020 (155) | COVID-19 | Global | Digital: Big Data, user input apps | Synthesis | Non-systematic literature review |
|  | Rimpiläinen, 2020 (156) | COVID-19 | Global | Digital: Big Data, user input apps | Synthesis | Non-systematic literature review |
|  | Zhang, 2022 (157) | COVID-19 | Global | Digital: Big Data, user input apps | Synthesis | Non-systematic literature review |
|  | Gunasekeran, 2021 (158) | COVID-19 | Global | Digital: Big data, user input apps, AI | Synthesis | Scoping review |
|  | Mbunge, 2020 (159) | COVID-19 | China | Digital: Big Data, user input apps, AI | Synthesis | Systematic review |
|  | Wu, 2020 (160) | COVID-19 | Germany | Digital: Big data, user input apps | Synthesis | Non-systematic literature review |
|  | Mitze, 2022 (161) | COVID-19 | USA | Digital: Infodemiology | Single study | Time series |
|  | McDonald, 2021 (162) | COVID-19 | Finland | Digital: Infodemiology | Single study | Time series |
|  | Mukka, 2021 (163) | COVID-19 | USA | Digital: Infodemiology | Single study | Time series |
|  | Ma, 2021 (164) | COVID-19 | North America | Digital: Infodemiology | Single study | Time series |
|  | Fenton, 2021 (165) | COVID-19 | Portugal | Digital: Infodemiology | Single study | Time series |
|  | Paiva Montero, 2021 (166) | COVID-19 | Global | Digital: Infodemiology | Single study | Time series |
|  | Ding, 2021 (167) | COVID-19 | China | Digital: Infodemiology | Single study | Time series |
|  | Wang, 2021 (168) | COVID-19 | International (Australia, Brazil, France, India, Iran, Italy, South Africa, UK, USA) | Digital: Infodemiology | Single study | Time series |
|  | Rabiolo, 2021 (169) | COVID-19 | Gironde, France | Digital: Infodemiology | Single study | Time series |
|  | Gil-Jardine, 2021 (170) | COVID-19 | USA | Digital: Infodemiology | Single study | Time series |
|  | Husnayain, 2021 (171) | COVID-19 | Global | Digital: Infodemiology | Single study | Time series |
|  | Zolbanin, 2021 (172) | COVID-19 | Global | Digital: Infodemiology | Single study | Time series |
|  | Abbas, 2021 (173) | COVID-19 | USA | Digital: Infodemiology | Single study | Time series |
|  | Fulk, 2021 (174) | COVID-19 | Africa | Digital: Infodemiology | Single study | Time series |
|  | Espinosa, 2021 (175) | COVID-19 | Global | Digital: Infodemiology | Single study | Time series |
|  | Alvarez, 2021 (176) | COVID-19 | Buenos Aires, Argentina | Digital: Infodemiology | Single study | Time series |
|  | Kogan, 2021 (177) | COVID-19 | USA | Digital: Infodemiology | Single study | Time series |
|  | Lu, 2021 (178) | COVID-19 | International | Digital: Infodemiology | Single study | Time series |
|  | Hamoui, 2020 (179) | COVID-19 | Saudi Arabia | Digital: Infodemiology | Single study | Time series |
|  | Chandra, 2020 (180) | COVID-19 | Indonesia | Digital: Infodemiology | Single study | Time series |
|  | Lopreite, 2021 (181) | COVID-19 | Europe | Digital: Infodemiology | Single study | Time series |
|  | Nsoesie, 2020 (182) | COVID-19 | South Africa | Digital: Infodemiology | Single study | Time series |
|  | Guo, 2020 (183) | COVID-19 | USA | Digital: Infodemiology | Single study | Cross-sectional |
|  | Kurian, 2020 (184) | COVID-19 | USA | Digital: Infodemiology | Single study | Time series |
|  | Cousins, 2020 (185) | COVID-19 | USA | Digital: Infodemiology | Single study | Time series |
|  | Peng, 2020 (186) | COVID-19 | Wuhan, China | Digital: Infodemiology | Single study | Time series |
|  | Mavragani, 2020 (187) | COVID-19 | Global | Digital: Infodemiology | Single study | Time series |
|  | Ben, 2022 (188) | COVID-19 | Australia, China, UK, USA | Digital: Infodemiology | Single study | Time series |
|  | Zhou, 2021 (189) | COVID-19 | USA | Digital: Infodemiology | Single study | Time series |
|  | Santangelo, 2021 (190) | COVID-19 | Italy | Digital: Infodemiology | Single study | Time series |
|  | Ben, 2021 (191) | COVID-19 | Global | Digital: Infodemiology | Single study | Time series |
|  | Momynaliev, 2021 (192) | COVID-19 | Russia | Digital: Infodemiology | Single study | Time series |
|  | Whitfield, 2021 (193) | COVID-19 | North Carolina, USA | Digital: Infodemiology | Single study | Time series |
|  | Park, 2021 (194) | COVID-19 | South Korea | Digital: Infodemiology | Single study | Time series |
|  | Mangono, 2021 (195) | COVID-19 | USA | Digital: Infodemiology | Single study | Time series |
|  | Kelly-Reif, 2021 (196) | COVID-19 | USA | Digital: Infodemiology | Single study | Time series |
|  | Huang, 2021 (197) | COVID-19 | Shenzhen, China | Digital: Infodemiology | Single study | Time series |
|  | Sulyok, 2021 (198) | COVID-19 | Europe | Digital: Infodemiology | Single study | Time series |
|  | Hisada, 2020 (199) | COVID-19 | Hokkaido, Japan | Digital: Infodemiology | Single study | Time series |
|  | Younis, 2020 (200) | COVID-19 | USA | Digital: Infodemiology | Single study | Time series |
|  | Li, 2020 (201) | COVID-19 | China | Digital: Infodemiology | Single study | Time series |
|  | Dai, 2020 (202) | COVID-19 | China | Digital: Infodemiology | Single study | Time series |
|  | Valentin, 2021 (203) | COVID-19 | Global | Digital: Infodemiology | Single study | Time series |
|  | Venkatesh, 2020 (204) | COVID-19 | India | Digital: Infodemiology | Single study | Time series |
|  | Rajan, 2020 (205) | COVID-19 | USA | Digital: Infodemiology | Single study | Time series |
|  | Buonanno, 2020 (206) | COVID-19 | Italy | Digital: Infodemiology | Single study | Modelling s |
|  | Higgins, 2020 (207) | COVID-19 | Global | Digital: Infodemiology using AI | Single study | Time series |
|  | Cai, 2021 (208) | COVID-19 | Global | Digital: Infodemiology using AI | Single study | Time series |
|  | Liu, 2021 (209) | COVID-19 | North Carolina, USA | Digital: Infodemiology using AI | Single study |  |
|  | Elgazzar, 2021 (210) | COVID-19 | Global | Digital: Infodemiology using AI | Single study | Cross-sectional |
|  | Alharbi, 2021 (211) | COVID-19 | Global | Digital: Infodemiology using AI | Single study | Time series |
|  | Lampos, 2021 (212) | COVID-19 | International | Digital: Infodemiology using AI | Single study | Time series |
|  | Lwowski, 2020 (213) | COVID-19 | Global | Digital: Infodemiology using AI | Single study | Modelling |
|  | Jiang, 2022 (214) | COVID-19 | USA | Digital: Infodemiology using AI | Single study | Time series |
|  | Peng, 2021 (215) | COVID-19 | Global | Digital: Infodemiology using AI | Single study | Cross-sectional |
|  | Wen, 2021 (216) | COVID-19 | Minnesota, USA | Digital: Infodemiology using AI | Single study | Time series |
|  | Shen, 2020 (217) | COVID-19 | China | Digital: Infodemiology using AI | Single study | Time series |
|  | Guo, 2021 (218) | COVID-19 | Global | Digital: Infodemiology using AI | Synthesis | Scoping review |
|  | Khemasuwan, 2021 (219) | COVID-19 | Global | Digital: Infodemiology using AI | Synthesis | Non-systematic literature review |
|  | Bragazzi, 2020 (220) | COVID-19 | Global | Digital: Infodemiology using AI | Synthesis | Non-systematic literature review |
|  | Urbaczewski, 2020 (221) | COVID-19 | China, Germany, Italy, Singapore, South Korea, USA | Digital: Passive app | Single study | Modelling |
|  | Weiss, 2021 (222) | COVID-19 | Brazil, China, Finland, France, Germany, Italy, Singapore, South Korea, Spain, UK | Digital: Passive app | Synthesis | Non-systematic literature review |
|  | Wirth, 2020 (223) | COVID-19 | Global | Digital: Passive app | Synthesis | Scoping review |
|  | Chowdhury, 2020 (224) | COVID-19 | Global | Digital: Passive app | Synthesis | Non-systematic literature review |
|  | ECDC, 2020 (225) | COVID-19 | Global | Digital: Passive app, user input app | Synthesis | Non-systematic literature review |
|  | Hurtado, 2021 (226) | COVID-19 | Texas and New York, USA | Digital: User input app | Single study | Time series |
|  | Van Dijk, 2021 (227) | COVID-19 | Netherlands | Digital: User input app | Single study | Time series |
|  | Guemes, 2021 (228) | COVID-19 | USA | Digital: User input app | Single study | Cross-sectional |
|  | Varsavsky, 2020 (229) | COVID-19 | England | Digital: User input app | Single study | Modelling |
|  | Zens, 2020 (230) | COVID-19 | Germany | Digital: User input app | Single study | Cross-sectional |
|  | Mehl, 2020 (231) | COVID-19 | Germany, UK | Digital: User input app | Single study | Time series |
|  | Quinn, 2021 (29) | COVID-19 | Global | Digital: User input app | Synthesis | Systematic review |
|  | Kennedy, 2021 (232) | COVID-19 | Sweden | Digital: User input app | Single study | Cohort |
|  | Sudre, 2020 (233) | COVID-19 | Israel, UK, USA | Digital: User input app and survey | Single study | Cohort |
|  | Drew, 2020 (234) | COVID-19 | UK, USA | Digital: User input app and survey | Single study | Time series |
|  | Chan, 2020 (235) | COVID-19 | Global | Digital: User input app and survey | Synthesis | Non-systematic literature review |
|  | Canas, 2021 (236) | COVID-19 | UK | Digital: User input app using AI | Single study | Cohort |
|  | Yom-Tov, 2021 (237) | COVID-19 | Israel | Digital: User input chat bot | Single study | Cohort |
|  | Martin, 2020 (238) | COVID-19 | Europe | Digital: User input chat bot using AI | Single study | Cohort |
|  | Yoneoka, 2020 (239) | COVID-19 | Tokyo | Digital: User input chat bot | Single study | Cohort |
|  | Maharaj, 2022 (240) | COVID-19 | Ontario, Canada | Digital: User input survey | Single study | Time series |
|  | McDonald, 2021 (241) | COVID-19 | Netherlands | Digital: User input survey | Single study | Cross-sectional |
|  | Salomon, 2021 (242) | COVID-19 | USA | Digital: User input survey | Single study | Cross-sectional |
|  | Runkle, 2021 (243) | COVID-19 | Buncombe County, North Carolina, USA | Digital: User input survey | Single study | Cross-sectional |
|  | Ku, 2020 (244) | COVID-19 | USA | Digital: User input survey | Single study | Cross-sectional |
|  | Leal-Neto, 2020 (245) | COVID-19 | Caruaru, Brazil | Digital: User input survey | Single study | Time series |
|  | Hegde, 2020 (246) | COVID-19 | Global | Digital: User input survey | Single study | Cross-sectional |
|  | Luo, 2020 (247) | COVID-19 | China | Digital: User input survey | Single study | Cross-sectional |
|  | Gong, 2020 (43) | COVID-19 | Honghu, China | Digital: User input survey | Single study | Cross-sectional |
|  | Wittwer, 2022 (248) | COVID-19 | Brazil | Digital: User input survey | Single study | Cohort |
|  | Tozzi, 2021 (249) | COVID-19 | Italy | Digital: User input survey | Single study | Time series |
|  | Reza, 2020 (250) | COVID-19 | Jacksonville and Duval County, Florida, USA | Digital: User input survey | Single study | Time series |
|  | Astley, 2021 (251) | COVID-19 | Global | Digital: User input survey with AI | Single study | Time series |
|  | Gu, 2022 (252) | COVID-19 | Hong Kong | Genomic | Single study | Cross-sectional |
|  | Walker, 2021 (253) | COVID-19 | Düsseldorf, Germany | Genomic | Single study | Cross-sectional |
|  | Nicholls, 2020 (254) | COVID-19 | UK | Genomic | Single study | Cross-sectional |
|  | Van Dorp, 2021 (255) | COVID-19 | Global | Genomic | Synthesis | Non-systematic literature review |
|  | Willis, 2021 (256) | COVID-19 | Massachusetts, USA | Health care setting | Single study | Time series |
|  | Boyle, 2022 (257) | COVID-19 | Queensland and South Australia, Australia | Health care setting | Single study | Time series |
|  | Boender, 2021 (258) | COVID-19 | Germany | Health care setting | Single study | Time series |
|  | Postill, 2022 (259) | COVID-19 | Ontario, Canada | Health care setting | Single study | Time series |
|  | Bouchouar, 2021 (260) | COVID-19 | Yukon, Canada | Health care setting | Single study | Time series |
|  | Papadomanolakis-Pakis, 2021 (261) | COVID-19 | Kingston, Frontenac and Lennox & Addington Health Region, Ontario, Canada | Health care setting | Single study | Time series |
|  | Stone, 2021 (262) | COVID-19 | USA | Health care setting | Single study | Cross-sectional |
|  | Ageron, 2021 (263) | COVID-19 | Vaud, Switzerland | Health care setting | Single study | Time series |
|  | Sayers, 2020 (264) | COVID-19 | Montgomery County, Maryland, USA | Health care setting | Single study | Time series |
|  | Pulia, 2020 (265) | COVID-19 | Midwestern USA | Health care setting | Single study | Time series |
|  | Saeed, 2022 (266) | COVID-19 | Global | Health care setting | Synthesis | Scoping review |
|  | Jenkins, 2020 (52) | COVID-19 | Hopi Tribe, USA | Indigenous methods | Single study | Cross-sectional |
|  | Amman, 2022 (267) | COVID-19 | Austria | Wastewater | Single study | Modelling |
|  | Lundy, 2021 (268) | COVID-19 | Global | Wastewater | Single study | Cross-sectional |
|  | Agrawal, 2021 (269) | COVID-19 | Europe | Wastewater | Single study | Time series |
|  | Hart, 2020 (270) | COVID-19 | Global | Wastewater | Single study | Modelling |
|  | Sharara, 2021 (271) | COVID-19 | Global | Wastewater | Synthesis | Non-systematic literature review |
|  | Mainardi, 2021 (272) | COVID-19 | Global | Wastewater | Synthesis | Non-systematic literature review |
|  | Wade, 2022 (273) | COVID-19 | UK | Wastewater | Synthesis | Non-systematic literature review |
|  | Shah, 2022 (274) | COVID-19 | Global | Wastewater | Synthesis | Systematic review |
|  | McClary-Gutierrez, 2021 (275) | COVID-19 | Global | Wastewater | Synthesis | Non-systematic literature review |
|  | Pena-Guzman, 2021 (276) | COVID-19 | Global | Wastewater | Synthesis | Non-systematic literature review |
|  | Amereh, 2021 (277) | COVID-19 | Global | Wastewater | Synthesis | Systematic review |
|  | Pulicharla, 2021 (278) | COVID-19 | Global | Wastewater | Synthesis | Non-systematic literature review |
|  | Olesen, 2021 (279) | COVID-19 | Global | Wastewater | Synthesis | Non-systematic literature review |
|  | Zechman Berglund, 2021 (280) | COVID-19 | Global | Wastewater | Synthesis | Non-systematic literature review |
|  | Donia, 2021 (281) | COVID-19 | Global | Wastewater | Synthesis | Non-systematic literature review |
|  | Li, 2021 (282) | COVID-19 | Global | Wastewater | Synthesis | Systematic review |
|  | Tiwari, 2021 (283) | COVID-19 | Global | Wastewater | Synthesis | Non-systematic literature review |
|  | Sharma, 2021 (284) | COVID-19 | Global | Wastewater | Synthesis | Non-systematic literature review |
|  | Zhu, 2021 (285) | COVID-19 | Global | Wastewater | Synthesis | Non-systematic literature review |
|  | Medema, 2020 (286) | COVID-19 | Global | Wastewater | Synthesis | Non-systematic literature review |
|  | Polo, 2020 (287) | COVID-19 | Global | Wastewater | Synthesis | Non-systematic literature review |
|  | Mandal, 2020 (288) | COVID-19 | Global | Wastewater | Synthesis | Non-systematic literature review |
|  | Dzinamarira, 2022 (289) | COVID-19 | Africa | Wastewater | Synthesis | Non-systematic literature review |
|  | Hrudey, 2021 (290) | COVID-19 | Global | Wastewater | Synthesis | Non-systematic literature review |
|  | Alhama, 2021 (291) | COVID-19 | Global | Wastewater | Synthesis | Non-systematic literature review |
|  | Aguiar-Oliveira, 2020 (292) | COVID-19 | Global | Wastewater | Synthesis | Non-systematic literature review |
|  | Anand, 2022 (293) | COVID-19 | Global | Wastewater | Synthesis | Systematic review |
|  | Kilaru, 2021 (30) | COVID-19 | Global | Wastewater | Synthesis | Systematic review |
|  | Corpuz, 2020 (294) | COVID-19 | Global | Wastewater | Synthesis | Non-systematic literature review |
|  | Bibby, 2021 (295) | COVID-19 | Global | Wastewater | Synthesis | Non-systematic literature review |
|  | Hill, 2021 (296) | COVID-19 | Global | Wastewater | Synthesis | Non-systematic literature review |
|  | Keshaviah, 2021 (297) | COVID-19 | USA | Wastewater | Synthesis | Non-systematic literature review |
|  | Ji, 2021 (298) | COVID-19 | Global | Wastewater | Synthesis | Scoping review |
|  | Anand, 2021 (299) | COVID-19 | Global | Wastewater | Synthesis | Non-systematic literature review |
|  | Hamouda, 2021 (300) | COVID-19 | Global | Wastewater | Synthesis | Non-systematic literature review |
|  | Kweinor Tetteh, 2020 (301) | COVID-19 | Global | Wastewater | Synthesis | Non-systematic literature review |
|  | Farkas, 2020 (302) | COVID-19 | Global | Wastewater | Synthesis | Non-systematic literature review |
|  | Abdeldayem, 2022 (303) | COVID-19 | Global | Wastewater using AI | Synthesis | Non-systematic literature review |
