# Supplementary material for: Changes to Public Health Surveillance Methods Due to the COVID-19 Pandemic: Scoping Review
Source: JMIR Public Health Surveill. 2024 Jan 19;10:e49185. doi: 10.2196/49185 (PMC10837764; doi:10.2196/49185)
Supplement: Multimedia Appendix 2 [file publichealth_v10i1e49185_app2.docx]

**Table A2: Included articles that describe implications of emerging public health surveillance methods**

|  | | | | **Perspectives** | | | |
| --- | --- | --- | --- | --- | --- | --- | --- |
| **Article** | **Type of article** | **Surveillance method(s)** | **Context** | **Ethics** | **Security** | **Legality** | **Equity** |
|  |  |  |  |  |  |  |  |
| Afroogh, 2021 (304) | Systematic review | Digital surveillance | Global | X |  |  |  |
| Ahn, 2021 (305) | Critical review | Digital surveillance | South Korea | X | X |  |  |
| Akhigbemen, 2020 (306) | Editorial | Digital surveillance | Global | X | X |  |  |
| Almeida, 2020 (307) | Editorial | Digital surveillance | Global | X | X |  |  |
| Andrei, 2020 (308) | Newspaper editorial | Digital surveillance | Moscow, Russia | X |  |  |  |
| Arriagada, 2020 (309) | Critical review | Digital surveillance | Global | X |  |  | X |
| Bagchi, 2020 (310) | Critical review | Digital surveillance | USA |  | X |  | X |
| Barriga, 2020 (311) | Critical review | Digital surveillance | Global | X |  |  |  |
| Berman, 2020 (312) | Critical review | Digital surveillance | Global | X |  |  |  |
| Bernot, 2021 (313) | Critical review | Digital surveillance | China | X |  |  |  |
| Bhardwaj, 2020 (314) | Critical review | Digital surveillance | India | X |  | X |  |
| Bradford, 2020 (315) | Critical review | Digital surveillance | USA |  |  | X |  |
| Calvo, 2020 (316) | Editorial | Digital surveillance | Global | X |  |  |  |
| Cofone, 2021 (317) | Critical review | Digital surveillance | Global | X | X |  | X |
| Correia, 2021 (318) | Critical review | Digital surveillance | Global | X |  | X |  |
| Couch, 2020 (319) | Critical review | Digital surveillance | Global | X |  |  |  |
| Cox, 2020 (320) | Editorial | Digital surveillance | Global | X |  |  |  |
| Csernatoni, 2020 (321) | Critical review | Digital surveillance | Global | X |  |  |  |
| Do Val da Fonseca, 2021 (322) | Critical review | Digital surveillance | Global | X |  |  |  |
| Eck, 2020 (323) | Critical review | Digital surveillance | Global | X |  |  |  |
| Elgujja, 2021 (324) | Critical review | Digital surveillance | Saudi Arabia |  | X | X |  |
| Foster, 2020 (325) | Critical review | Digital surveillance | USA |  |  | X |  |
| Gasser, 2020 (326) | Critical review | Digital surveillance | Global | X | X | X | X |
| Goldenfein, 2020 (327) | Newspaper Editorial | Digital surveillance | Global | X |  |  |  |
| Gomez-Ramirez, 2021 (328) | Critical review | Digital surveillance | Global | X |  |  | X |
| Greenleaf, 2020 (329) | Critical review | Digital surveillance | Australia |  | X | X |  |
| Greenleaf, 2021 (330) | Newspaper editorial | Digital surveillance | Australia | X |  | X |  |
| Greitens, 2020 (331) | Editorial | Digital surveillance | Global | X |  |  |  |
| Hendl, 2020 (332) | Critical review | Digital surveillance | Global | X |  |  | X |
| Holmes, 2020 (333) | Critical review | Digital surveillance | USA |  |  | X |  |
| Huddleston, 2020 (334) | Newspaper Editorial | Digital surveillance | Global | X |  |  |  |
| Ienca, 2020 (335) | Editorial | Digital surveillance | Global | X | X |  |  |
| Kampmark, 2020 (336) | Critical review | Digital surveillance | Global | X |  |  |  |
| Khan, 2021 (337) | Editorial | Digital surveillance | Global | X |  |  |  |
| Kitchin, 2020 (338) | Editorial | Digital surveillance | Global | X |  |  |  |
| Kliestik, 2021 (339) | Critical review | Digital surveillance | Global |  |  | X |  |
| Kokal, 2020 (340) | Newspaper Editorial | Digital surveillance | India | X |  |  | X |
| Kui, 2021 (341) | Critical review | Digital surveillance | China | X |  | X |  |
| Leonov, 2020 (342) | Editorial | Digital surveillance | Global | X |  |  |  |
| Marquez Carrasco, 2020 (343) | Critical review | Digital surveillance | Spain |  |  | X | X |
| Maati, 2021 (344) | Editorial | Digital surveillance | Global | X |  |  |  |
| Mann, 2020 (345) | Critical review | Digital surveillance | Global | X | X |  |  |
| Mavriki, 2020 (346) | Critical review | Digital surveillance | Global | X | X |  |  |
| McGrail, 2021(347) | Editorial | Digital surveillance | Global | X |  |  | X |
| Miller, 2021 (348) | Critical review | Digital surveillance | Global | X | X |  |  |
| Oliva, 2020 (349) | Critical review | Digital surveillance | USA |  |  | X |  |
| Oliva, 2021 (350) | Critical review | Digital surveillance | USA |  |  | X |  |
| Roberts, 2020 (351) | Editorial | Digital surveillance | Global | X |  |  |  |
| Rule, 2020 (352) | Newspaper Editorial | Digital surveillance | USA | X |  |  |  |
| Seto, 2021 (353) | Editorial | Digital surveillance | Global | X |  |  |  |
| Sharon, 2020 (354) | Critical review | Digital surveillance | Global | X |  |  |  |
| Shen, 2021 (355) | Critical review | Digital surveillance | China | X |  | X |  |
| Subbian, 2021 (356) | Editorial | Digital surveillance | USA | X |  |  |  |
| Sundquist, 2021 (39) | Critical review | Digital surveillance | Global |  |  |  | X |
| Tewari, 2021 (357) | Editorial | Digital surveillance | Global | X |  |  |  |
| Timotijevic, 2020 (358) | Editorial | Digital surveillance | Global | X |  |  |  |
| Tromblay, 2021 (359) | Editorial | Digital surveillance | USA | X | X |  |  |
| Unger, 2020 (360) | Critical review | Digital surveillance | Global | X | X |  |  |
| Venkatasubramanian, 2020 (361) | Editorial | Digital surveillance | Global | X |  |  |  |
| Vitak, 2020 (362) | Editorial | Digital surveillance | Global | X |  |  |  |
| Wee, 2020 (363) | Critical review | Digital surveillance | Australia, Taiwan | X |  | X |  |
| Yang, 2020 (364) | Critical review | Digital surveillance | China, South Korea, USA | X |  |  |  |
| Yu, 2020 (365) | Critical review | Digital surveillance | Global | X |  |  |  |
| Yuniarti, 2021 (366) | Critical review | Digital surveillance | Indonesia | X |  |  |  |
| Zhao, 2021 (367) | Systematic Review | Digital surveillance | Global | X | X |  |  |
| Zinn, 2021 (368) | Critical review | Digital surveillance | Global | X | X | X | X |
| Xu, 2020 (369) | Critical review | Digital surveillance | Global |  |  | X |  |
| Calvo, 2020 (316) | Editorial | Digital surveillance | Global | X |  |  |  |
| Bentotahewa, 2021 (370) | Critical review | Digital surveillance | Global | X | X |  |  |
| Cassiano, 2021 (371) | Critical review | Digital surveillance | China |  |  |  |  |
| Frith, 2020 (372) | Critical review | Digital surveillance | Global | X | X |  | X |
| Mesarcik, 2020 (373) | Critical review | Digital surveillance | Slovakia | X | X |  |  |
| Rashied Hussein, 2020a (374) | Critical review | Digital surveillance | Global |  | X |  |  |
| Rashied Hussein, 2020b (375) | Critical review | Digital surveillance | Global | X | X | X |  |
| Ramjee, 2020 (376) | Critical review | Digital surveillance | USA |  | X | X |  |
| Cong, 2021 (377) | Critical review | Digital surveillance | China | X | X |  |  |
| Pawlotsky, 2020 (378) | Critical review | Digital surveillance | Global | X |  |  |  |
| Ryan, 2020 (379) | Critical review | Digital surveillance | South Korea | X |  |  |  |
| Veena, 2020 (380) | Newspaper Editorial | Digital surveillance | Global | X |  |  | X |
| Li, 2020 (381) | Critical review | Digital surveillance | Global |  |  | X |  |
| Liu, 2021 (382) | Critical review | Digital surveillance | China | X |  |  |  |
| Lucivero, 2020 (383) | Critical review | Digital surveillance | Global | X |  |  |  |
| Madianou, 2020 (384) | Editorial | Digital surveillance | Global |  |  |  | X |
| Mello, 2020 (385) | Editorial | Digital surveillance | Global | X | X |  |  |
| Nabben, 2020 (386) | Critical review | Digital surveillance | Global | X |  |  |  |
| Newlands, 2020 (387) | Critical review | Digital surveillance | Global | X |  |  |  |
| Ngan, 2021 (388) | Editorial | Digital surveillance | Global | X |  |  |  |
| Ram, 2020 (389) | Critical review | Digital surveillance | USA | X |  | X |  |
| Ramesh, 2020 (390) | Critical review | Digital surveillance | Global | X | X |  | X |
| Roberts, 2020 (391) | Editorial | Digital surveillance | Global | X |  |  |  |
| Rozenshtein, 2020 (392) | Critical review | Digital surveillance | USA | X |  | X |  |
| Sharifah, 2020 (393) | Critical review | Digital surveillance | Global | X |  |  |  |
| Spears, 2021 (394) | Critical review | Digital surveillance | Global | X | X |  | X |
| Surber, 2020 (395) | Editorial | Digital surveillance | Global | X |  | X |  |
| Scassa, 2020 (396) | Critical review | Digital surveillance with AI | Global | X |  |  |  |
| Von Struensee, 2021 (397) | Critical review | Digital surveillance with AI | Global | X |  | X | X |
| Mahapatra, 2021 (398) | Editorial | Digital surveillance with AI, social media | India | X |  |  |  |
| Dawe, 2021 (399) | Literature review | Digital surveillance, infodemiology | Global | X |  |  |  |
| Dayalani, 2020 (400) | e-Book: Literature review | Digital surveillance, infodemiology | Global | X |  |  |  |
| Bariffi, 2021 (401) | Critical review | Digital surveillance, infodemiology, with AI | Global | X |  | X |  |
| Zarra, 2021 (402) | Editorial | Digital with AI | Europe |  |  | X | X |
| Findlay, 2020a (403) | Critical review | Digital with AI | Singapore, China | X | X |  | X |
| Findlay, 2020b (404) | Critical review | Digital with AI | Global | X | X | X | X |
| Leslie, 2020 (405) | Editorial | Digital with AI | Global | X |  |  | X |
| Carmel, 2020 (406) | Critical review | Infodemiology with AI | Global | X | X | X |  |
| Gable, 2020 (407) | Editorial | CDC data | USA | X |  |  | X |
| Honda, 2021 (408) | Critical review | Wastewater | Global | X |  | X | X |
| Hrudey, 2021 (409) | Critical review | Wastewater | Global | X |  |  | X |
| Joh, 2020 (410) | Critical review | Wastewater | Canada | X |  |  |  |
